# Choledochoscopic high-frequency needle-knife electrotomy for treatment of anastomotic strictures after Roux-en-Y hepaticojejunostomy

**DOI:** 10.1186/s12876-016-0465-9

**Published:** 2016-05-06

**Authors:** Yu-long Yang, Cheng Zhang, Ping Wu, Yue-feng Ma, Jing-yi Li, Hong-wei Zhang, Li-jun Shi, Mei-ju Lin, Ying Yu

**Affiliations:** Department of Biliary Minimally Invasive Surgery, Affiliated Zhongshan Hospital of Dalian University, No, 6. Jiefang Road, Zhongshan District, Dalian, Liaoning Province 116001 P. R. China

**Keywords:** Anastomotic stricture, Hepaticojejunostomy, Balloon dilatation, Electrotomy, Choledochoscope

## Abstract

**Background:**

Anastomotic stricture is a complex and substantial complication following Roux-en-Y hepaticojejunostomy. Initially, endoscopic and percutaneous approaches are often attempted, but the gold standard remains surgical biliary reconstruction, especially for refractory stricture. However, this solution leaves much room for improvement, due to the challenging nature of the biliary reconstruction procedure, in which anastomotic stricture may still occur.

**Aims:**

To investigate the feasibility and effectiveness of choledochoscopic high-frequency needle-knife electrotomy as an intervention in the treatment of anastomotic strictures following Roux-en-Y hepaticojejunostomy.

**Methods:**

From February 2010 to October 2014, clinical data was collected and retrospectively compared for patients who underwent balloon dilation or/and choledochoscopic high-frequency needle-knife electrotomy for the treatment of anastomotic strictures after Roux-en-Y hepaticojejunostomy.

**Results:**

A total of 38 patients underwent successful choledochoscopic treatment and all the anastomotic strictures were removed successfully, 19 of which were treated with electrotomy, 7 with balloon dilation, and 12 with both electrotomy and balloon dilation. Among these groups,the average operating times were 6.9 ± 2.4 min,10.1 ± 6.8 min, and 20.2 ± 13.5 min, respectively. The average stent supporting times were 6.3 ± 0.7 months, 6.5 ± 0.6 months, and 6.1 ± 0.4 respectively. The mean follow-up after stent removal was 42.1 ± 27.4 months, and in 26.3 % (5/19), 28.5 % (2/7) and 16.7 % (2/12) of cases, recurrent anastomotic stricture occurred. Of these 9 total patients with recurrent anastomotic, two patients were successfully rescued by full-covered self-expanding removable metal stents and 7 patients by electrotomy combined with balloon dilation.

**Conclusions:**

Choledochoscopic high-frequency needle-knife electrotomy is both feasible and safe in the treatment of anastomotic stricture after Roux-en-Y hepaticojejunostomy, with a similar long-term outcome to balloon dilation in treating anastomotic stricture after Roux-en-Y hepaticojejunostomy. A combination of choledochoscopic electrotomy concurrent with balloon dilation should be recommended based on the low rate of recurrence.

## Background

Roux-en-Y hepaticojejunostomy is a common surgical procedure used to by-pass extrahepatic biliary obstructions and to establish biliary-enteric continuity after resections for benign and malignant diseases [1], such as choledochal cysts, post-cholecystectomy bile ducts injuries, and periampullary carcinoma [2, 3]. Multiple studies examining the long-term outcome of biliary reconstruction showed that Roux-en-Y hepaticojejunostomy is feasible and safe with contained morbidity and durable results [4, 5]. However, some postoperative conditions are still unavoidable, including intrahepatic lithiasis, reflux cholangitis, and biliary anastomotic stricture (AS) [6]. Of these, biliary-enteric anastomotic stricture occurred at a rate of 6.87 % at 2-13 years follow-up, and is accompanied by significant inducement of other complications [7].

Surgical biliary reconstruction is still the main approach and the gold standard in treating biliary stricture after Roux-en-Y hepaticojejunostomy, especially for the treatment of refractory stricture. Although good results may be achieved up to twice in the form of Roux-en-Y hepaticojejunostomy [8], it would be extremely difficult to manage such biliary reconstruction in patients with complicated cases, and AS may still occur, regardless of whether or not a stent is used [9]. In this situation, the initial approach for benign stricture of the anastomosis after hepaticojejunostomy has often been to use promising, minimally invasive procedures, such as the endoscopic and percutaneous approaches. As a first step, or even as definitive management in treating these patients, an ultrasonic or fluoroscopic guided percutaneous transhepatic cholangiography (PTC) procedure is performed, followed by choledochoscopic lithotomy, balloon dilatation, and long-term internal/external drainage [10]. Full-covered self-expanding removable metal stents may be used for persistent strictures after transhepatic intubation [11].

Choledochoscopic high-frequency needle-knife electrotomy (CHFNKE) is a new technology developed from endoscopic sphincterotomy. We have previously reported it as a potential complementary medical approach for treating intrahepatic biliary stricture associated with hepatolithiasis. This alternative can be especially useful when the bile duct is obstructed with stones or when the diameter of the bile duct is narrow [12]. In the current study, we retrospectively compared the effects of long-term internal/external drainage following CHFNKE, balloon dilation (BD), or CHFNKE combined with BD, in terms of the technical complications and long-term outcome of AS after Roux-en-Y hepaticojejunostomy. Our data confirmed that the new CHFNKE procedure could be a potential complementary medical approach for treating anastomotic stricture.

## Methods

### Patients

From February 2010 to October 2014, 65 patients diagnosed with AS following Roux-en-Y hepaticojejunostomy were enrolled in the Department of Biliary Minimally Invasive Surgery, affiliated Zhongshan Hospital of Dalian University in China. AS was defined as the diameter of the anastomosis is less than 1/2 diameter of the bile duct above the anastomosis. Twenty-six patients with a T drainage tube had postoperative AS, 15 of these patients acquired AS after the first Roux-en-Y hepaticojejunostomy, and 11 after the second hepaticojejunostomy. Of the thirty-nine patients who did not have T tube drainage, 31 had postoperative AS following the first Roux-en-Y hepaticojejunostomy and 8 had recurrent AS after the second hepaticojejunostomy.

The patients eligible for inclusion were those who underwent endoscopic long-term internal/external drainage following one of the next choledochoscopic interventions via T tube fistulous tract: CHFNKE (electrotomy group), balloon dilation (balloon group), CHFNKE combined with BD (mixing group). Exclusion criteria were: 1) conservative treatment; 2) percutaneous transhepatic biliary drainage (PTBD) intervention; 3) placement of metal stents; 4) persist balloon dilation. All patients provided written informed consent prior to undergoing any procedures and informed consent was obtained from each patient.

### Clinical symptoms and preoperative examination

Most patients showed symptoms, especially after consuming greasy food, including recurrent episodes of subxiphoid pain, chills and fever, and jaundice. Preoperative workup included liver function tests (LFTs), which assess the levels of aspartate aminotransferase, alanine aminotransferase, alkaline phosphate, and total bilirubin. In addition to abdominal ultrasound, computerized tomography (CT) and magnetic resonance cholangiopancreatography (MRCP) or T tube cholangiography were performed as part of the preoperative assessment of strictures and stones.

### Choledochoscopic equipment and accessories

The following equipment and accessories were utilized during endoscopy: CYF-AV2 electron choledochoscope, CHF-XP20 and CHF-BP30 fiber cholangioscopes (Olympus, Japan), visualization catheter (Olympus, Japan), VIO-200 s high frequency generator (mixed currents, cut current of 40-W, coagulation current of 40-W) (ERBE, Germany), DLZ-2 plasma shock wave lithotripter (Edragon, Beijing), needle-knife (Endo-Flex, Germany), balloon dilatation catheter (balloon diameter, 6 to 12 mm; length, 4 cm; pressure, 8 to 18 ATM) (OptiMed, Germany), inflation device (Boston Scientific, USA), yellow zebra guide wire (Boston Scientific, USA), extraction basket (diameter, 10 mm; length, 115 cm) (COOK, USA), self-made external-internal biliary stent and built-in plastic stent with multiple side holes formed by T tube (Weishitai, China), and COOK drainage tube (COOK, USA).

### Surgical biliary exploration

The patients without T tube or subcutaneous access underwent surgical biliary exploration. After satisfactory local anesthesia or general anesthesia, the abdominal wall was cut and the output loop of the jejunum after Roux-en-Y hepaticojejunostomy was found. A 0.5 cm incision on the jejunum was made, through which a choledochoscope containing an adsorber at the end was placed in order to find the biliary intestinal anastomosis using the guide of bile. At the end of the operation, a T drainage tube was placed into the jejunum through the abdominal wall and four titanium clips were sutured into the jejunum wall surrounding the T drainage tube.

### Therapeutic choledochoscope

#### Choledochoscopy and cholangiography

A choledochoscope containing an adsorber at the end was inserted into jejunum via the T-tube fistulous tract and the biliary intestinal anastomosis was found with the guide of bile. In order to visualize bile duct morphology and the presence of possible stones, digital subtraction angiography was used by inserting a catheter into the common bile duct from the working channel of the choledochoscope, through which a contrast agent was injected. If the opening diameter of the anastomosis was significantly smaller than the distal bile duct, AS was confirmed.

### Choledochoscopic high-frequency needle-knife electrotomy

After confirmation of AS by choledochoscope and cholangiography, choledochoscopic high-frequency needle-knife electrotomy could be performed using a high frequency generator with a setting of mixed currents, with a cut current of 40-W and a coagulation current of 40-W. First, the end of the choledochoscope was fixed and the strictured opening was focused in the center of the visual fields. Then a high-frequency needle-knife was inserted into the restricted anastomosis via the working channel of choledochoscope until the head of needle-knife in the center of the visual fields. Usually, the head of needle-knife was located in 2 to 3 mm to the working channel opening of choledochoscope. In the next, the main power source was turned on and the head of choledochoscope was removed slowly to the root of hypertrophic scar tissue. Finally the strictured opening was cut or removed gradually until the root, under direct vision by blend current. Upon signs of bleeding at the strictured bile duct or jejunal wall, electrocoagulation would be performed to prevent further bleeding. It should be noted that the diameter of the anastomotic stoma after choledochoscopic high-frequency needle-knife electrotomy was not larger than that of the upper bile duct, and perforation would almost never happen.

### Choledochoscopic balloon dilatation

First, yellow zebra guide wire was inserted from the working channel of choledochoscope into the intrahepatic bile duct and through the AS. A 6 mm balloon dilatation catheter was then inserted under the guidance of the yellow zebra guide wire. The balloon was gradually inflated to the maximum pressure. After 5 min, the pressure was then gradually released, followed by refilling once. A larger balloon dilatation catheter would be used according to the degree of dilatation of the common or intrahepatic bile duct. If the strictured bile duct or jejunal wall tissue showed signs of bleeding, electrocoagulation or refilling balloon dilatation would be performed to prevent further bleeding.

### Removal of bile duct stones under choledochoscope

After the stricture was resolved, an extraction basket was inserted into the bile duct to remove stones. Smaller stones were drawn off through the adsorber at the end of the choledochoscope, while larger stones were shredded using a plasma shock wave lithotripter. Multiple choledochoscopic operations were performed until there were no residual stones visible by ultrasound, CT, and cholangiography.

### Insertion of supporting stents

In order to prevent stricture recurrence, either self-made external-internal biliary stents or built-in plastic stents with multiple side holes were inserted across the stricture under the guidance of yellow zebra guide wire. Postoperative choledochoscopy was performed every 2 to 3 months to replace the blocked stents. After more than 6 months, supporting stents could be removed, and subsequent choledochoscopy was performed two weeks after the stent removal.

### Qualifying standards for stent and drainage tube removal

(i) Mucosa of the anastomotic stricture repaired normally without edema and scar formation, (ii) No stricture. It was defined as the diameter of the AS is more than the diameter of the bile duct above the anastomosis (iii) No floc, sludge, or residual stones in the intrahepatic or extrahepatic bile duct.

### Follow-up

All patients received the same postoperative care provided by the same team of surgeons. Assessment of postoperative liver function and ultrasound were conducted every three to six months, and then annually, or whenever patients presented with symptoms suggestive of cholangitis. MRCP and CT were performed if ultrasound results indicated signs of recurrent stones or anastomotic strictures.

### Statistical analysis

Quantitative date were assessed by Student’s *t*-test. *P* < 0.05 was considered to be statistically significant. All statistical analyses were performed using SPSS software package, version 21 (Statistical Package for Social Sciences, IBM Corporation, Armonk, NY, USA).

## Results

### Patient characteristics

**Fig. 1:**
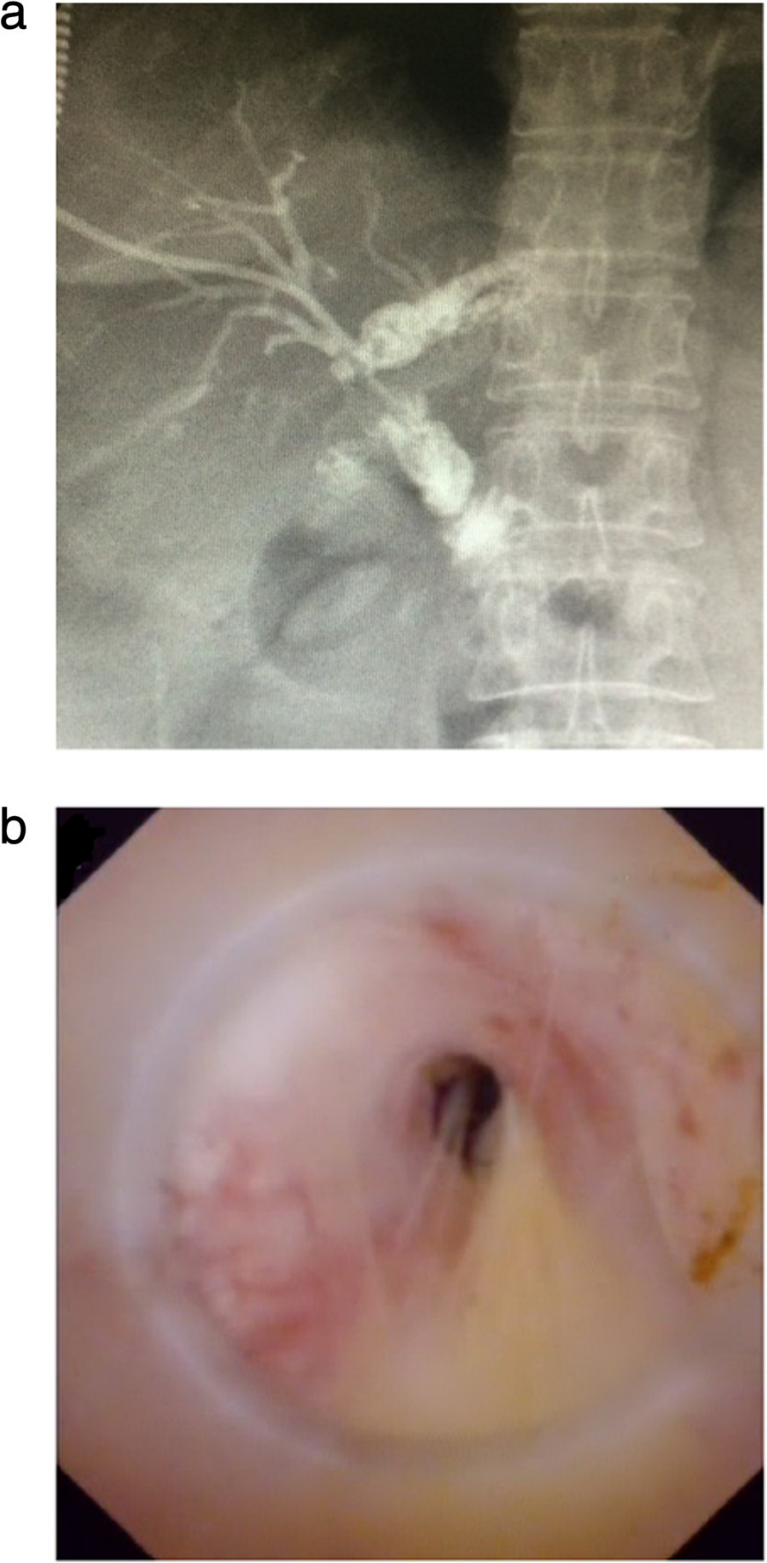
Anastomotic strictures after Roux-en-Y hepaticojejunostomy: **a**) Percutaneous transhepatic cholangiography showed the dilatation of the left hepatic duct and anastomotic stricture. **b**) A narrow opening of bile duct can be seen under choledochoscope

The present study included thirty-eight patients with AS after Roux-en-Y hepaticojejunostomy (Fig. 1). The age range of patients was 34 to 72 years, of those 15 patients were male and 23 were female. The causes of hepaticojejunostomy contained 8 cases of iatrogenic bile duct injury, 2 cases of traumatic bile duct injury, 2 cases of choledochal cysts, 17 cases for intrahepatic bile duct stones, 6 cases of suspicious pancreatic head carcinoma, 3 cases of serious reflux cholangitis after endoscopic sphincterotomy. In the 38 patients, only 18 patients had a T drainage tube, however, 7 patients had recurrent AS after a second hepaticojejunostomy. Eleven patients were diagnosed as tubular stricture, and 27 patients were diagnosed as membranous stricture. The average time after Roux-en-Y hepaticojejunostomy was 18.8 ± 6.6 months. Five patients presented with simple AS, but 33 patients presented with hepatolithiasis (Table 1).

**Table 1:** Characteristics of the 38 patients

	Number
Age (years)	49.6 ± 23.1
Range	34~72
Gender	
Male	15
Female	23
Causes of hepaticojejunostomy	
Iatrogenic bile duct injury	8
Traumatic bile duct injury	2
Choledochal cyst	2
Intrahepatic bile duct stones	17
Suspicious pancreatic head carcinoma	6
Reflux cholangitis after EST	3
With T drainage tube	
Yes	18
No	20
Hepaticojejunostomy	
Once	31
Twice	7
Stricture and hepatolithiasis	
Simple stricture	5
Stricture with hepatolithiasis	33
Stricture type	
Membranous stricture	27
Tubular stricture	11

### Operation procedure and complication characteristics

**Fig. 2:**
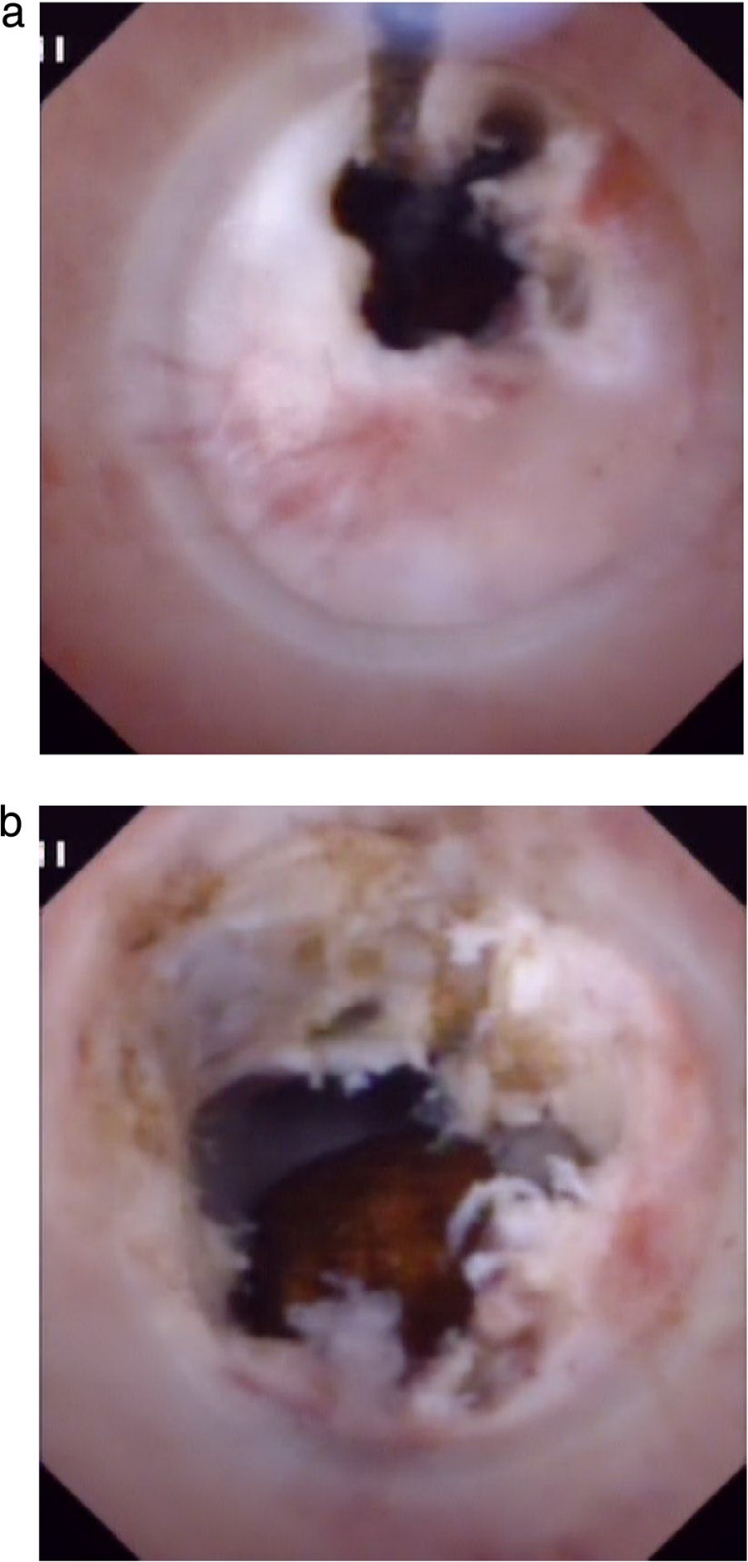
Choledochoscopic high-frequency needle-knife electrotomy was performed for anastomotic strictures: **a**) The strictured opening is cut gradually under direct vision with no sign of bleeding. **b**) The fibrous tissue of the anastomosis has been removed and a stone can be seen in intrahepatic bile duct

**Fig. 3:**
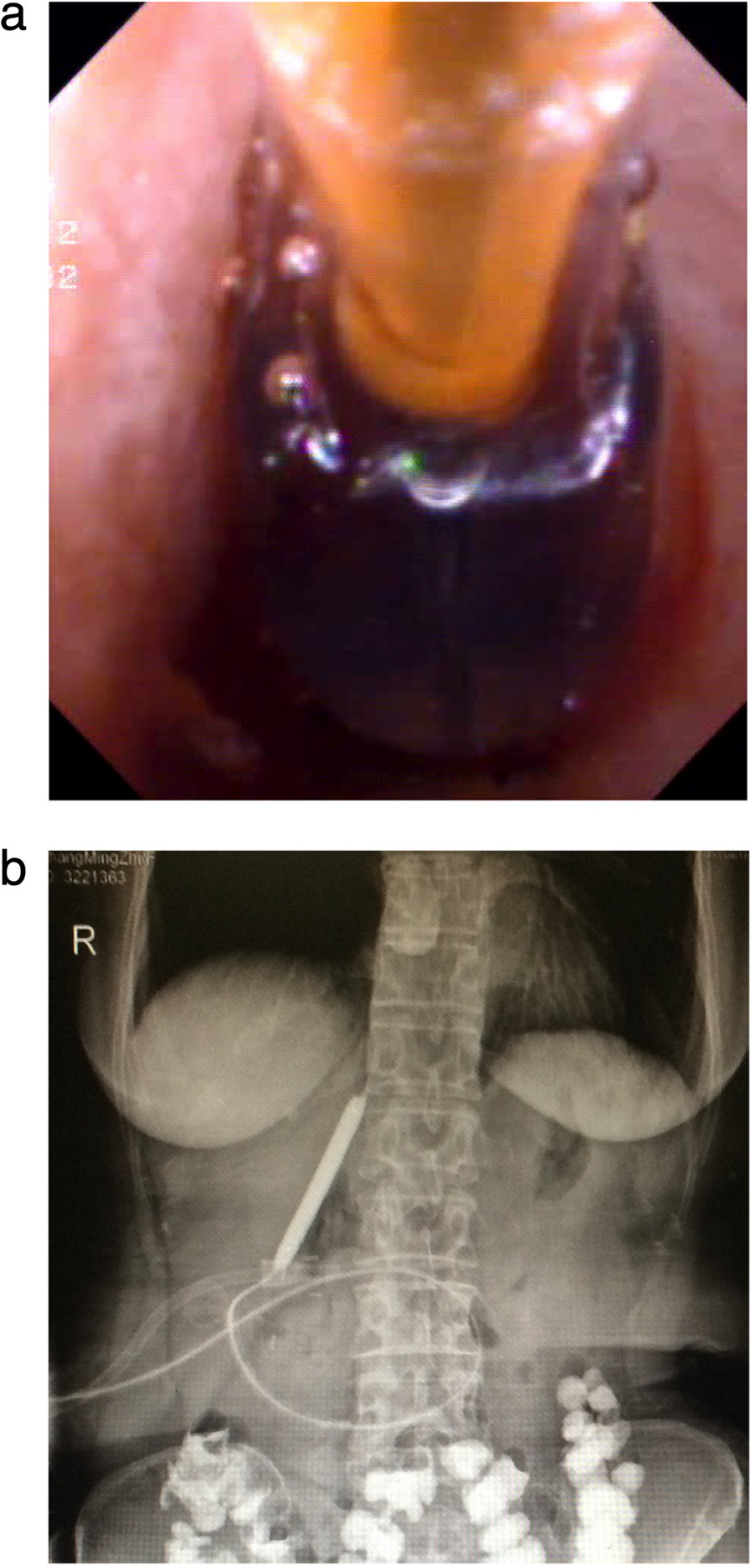
Balloon dilation of anastomotic strictures: **a**) Choledochoscopic balloon dilatation of the anastomotic stricture under the guidance of the yellow zebra guide wire. **b**) Balloon dilation under X-ray

Nineteen membranous strictures were treated with CHFNKE (Fig. 2), 7 tubular strictures with BD (Fig. 3), 8 membranous strictures and 4 tubular strictures with CHFNKE and BD . All the anastomotic strictures were removed successfully. The average operating time was 6.9 ± 2.4 min,10.1 ± 6.8 min, and 20.2 ± 13.5 min in electrotomy group, balloon group and mixing group (electrotomy group vs balloon group, *P* = 0.262; balloon group vs mixing group, respectively, *P* = 0.087; electrotomy group vs mixing group, *P* = 0.006). There were 2 cases of intraoperative hemorrhage in the balloon group, which were cured by choledochoscopic needle-knife electrocoagulation (Table 2).

**Table 2:** Operation procedure and complication characteristics

	Electrotomy group	Balloon group	Mixing group
Patients	19	7	12
Operating time (Minutes)	6.9±2.4	10.1±6.8	20.2±13.5
Complication			
Hemorrhage	0	2	0
Drainage time (Months)	6.3±0.7	6.5±0.6	6.1±0.4
Recurrent AS	26.3% (5/19)	28.5% (2/7)	16.7% (2/12

### Effectiveness of the operation

**Fig. 4:**
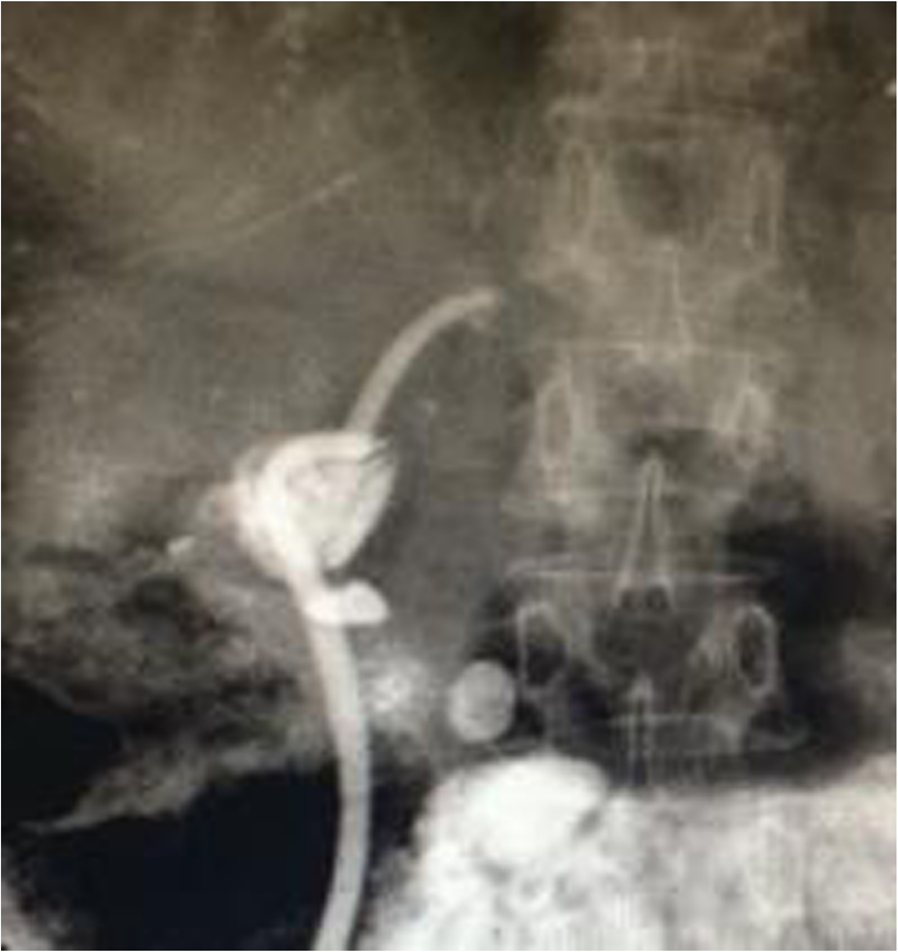
Self-made external-internal biliary stents and built-in plastic stents with multiple side holes were inserted across the stricture to prevent stricture recurrence

**Fig. 5:**
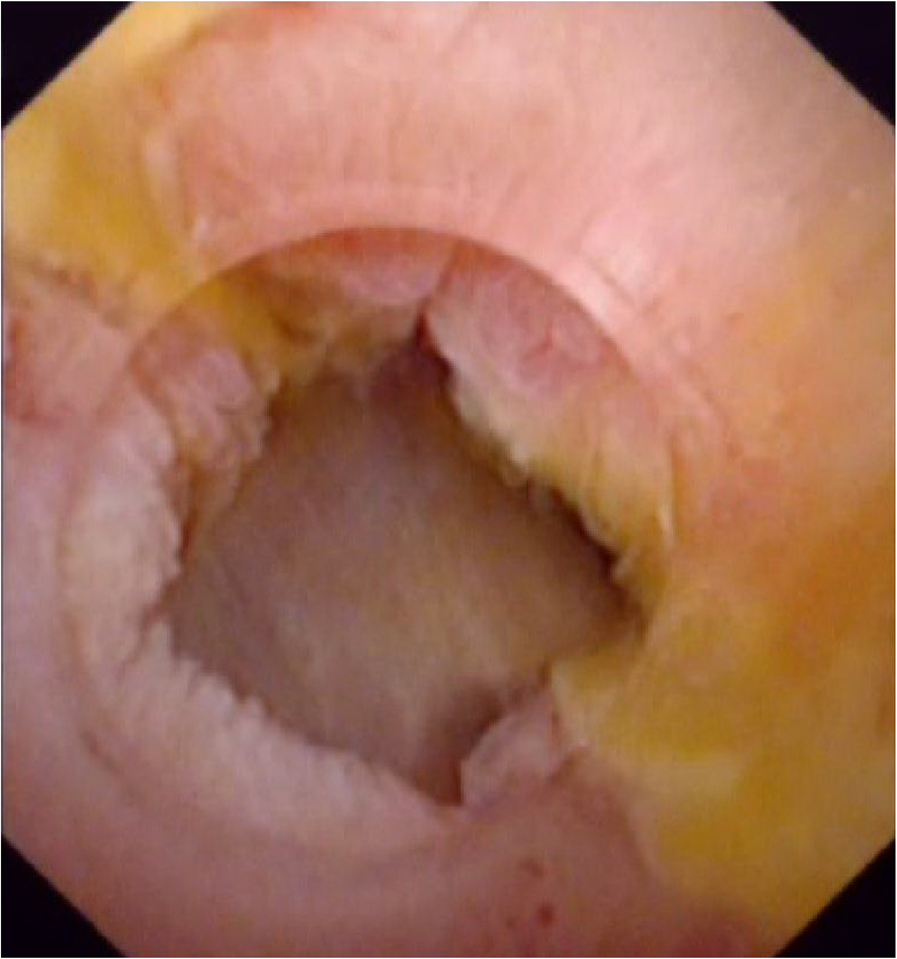
supporting stent has been removed after more than 6 months, and subsequent choledochoscopy shows the mucosa of the anastomotic stenosis repaired normally without edema and scar formation. There is no stricture, relative stricture, floc, sludge, or residual stone in extrahepatic bile duct

Among the three groups, the mean period of drainage catheter placement was 6.3 ± 0.7 months, 6.5 ± 0.6 months, and 6.1 ± 0.4 months (electrotomy group vs balloon group, *P* = 0.737; balloon group vs mixing group, respectively, *P* = 0.146; electrotomy group vs mixing group, *P* = 0.252) (Fig. 4). Endoscopic cholangiography revealed good patency of the biliary trees. Bile duct and jejunal mucosa around the incision recovered appropriately with no sign of AS or stones, as confirmed by choledochoscopy done two weeks after removal of the stents (Fig. 5).

### Follow-up result

With a mean follow-up of 42.1 ± 27.4 months (15 ~ 59 months) after removal of the drainage catheter, 26.3 % (5/19), 28.5 % (2/7), and 16.7 % (2/12) of patients presented with recurrent anastomotic stricture in electrotomy group, balloon group and mixing group, respectively (Table 2). Of the 9 recurrent patients, 2 patients were successfully rescued by full-covered self-expanding removable metal stents and 7 patients were rescued by CHFNKE and BD.

## Discussion

Although surgical hepaticojejunostomy plays an important role in the therapy for AS after Roux-en-Y hepaticojejunostomy, reduplicative biliary reconstruction remains a challenging topic for hepatobiliary surgeons, and a considerable percentage of patients still need endoscopic or intercurrent treatment. The standardized regimens in treating AS involve two operational steps: first, to remove the stricture;second, to prevent restricture. Because of this, balloon dilatation and long-term internal/external drainage become the primary approach and gold standard for minimally invasive procedures, especially for patients requiring multiple operations [13].

At present, BD is the main method to relieve AS, and there are several schemes of balloon dilatation, with differing results at follow-up. An early study reported that 93.3 % percent (14/15) of the strictures could be successfully dilated by the BD, but the incidence of restricture was high and could reach up to 45 % within a long-term follow-up period of 5 to 7 1/2 years [14]. Another research study showed that the success rate for one round of balloon dilatation was 73 % after a mean follow-up of 30 months, but when repeated, the success rate was 80 % after a mean follow-up of 33 months [15]. Moreover, long-term balloon dilatation following hepaticojejunostomy can improve the outcome of AS [16]. Collectively, these studies suggested that the increasing number and cumulative time of balloon dilatation could significantly improve the outcome of AS after Roux-en-Y hepaticojejunostomy, most likely because of tissue remodeling at the site of anastomosis during the period of wound healing. Therefore, in order to achieve an exceptionally high success rate, the placement of internal/external biliary drainage was recommended. If the AS was resolved by balloon dilatation, the biliary drainage should be inserted for 2 to 6 months, otherwise the duration recommended for insertion was 3 to 12 months [14, 17]. When compared with simple balloon dilatation, balloon dilatation in combination with internal/external biliary drainage increases the operation’s effect, with a one year primary clinical success rate of 94 % for the treatment for AS [18]. In our study, the mean period of drainage catheter placement was more than 6 months, and 73.7 % (14/19) of patients achieved success with a mean follow-up of 4 years.

As we know, intrahepatic duct stones and AS usually occur concurrently [17]. A vicious cycle exists between the presence of intrahepatic calculi and chronic proliferative cholangitis [19]. In the later stages of inflammation, a stricture of the duct may occur when inflammation and fibrosis are localized within a short segment of the duct [20]. When the bile duct is obstructed with stones or when the diameter of the bile duct is finer than balloon catheter, BD is usually difficult to be performed. In this situation, we invented CHFNKE technology. The effect of high temperature carbonization caused by CHFNKE could gradually remove the hyperplastic fibrous tissues around the anastomosis and enlarge the stoma easily. This technology has been proven to be a safe and highly effective approach in the treatment of intrahepatic biliary stricture,with a high rate of immediate stone clearance, a low rate of long-term stone recurrence, and importantly, a good long-term follow-up outcome [12]. In our study, the average operating time for relieving AS after Roux-en-Y hepaticojejunostomy was 6.9 ± 2.4 min,10.1 ± 6.8 min, and 20.2 ± 13.5 min in electrotomy group, balloon group and mixing group respectively. Although the mean operating time in electrotomy group was shorter than balloon group, there was no obvious difference between the two group (*P* = 0.262 > 0.05). The date further validated that the operation of CHFNKE was more difficult than BD, but the effect of chemical high temperature carbonization caused by CHFNKE was better than the mechanical tear caused by BD in the treatment of AS.

An increase in connective tissues could cause the wall of the duct to thicken and to show signs of fibrosis, proliferation of mucous and serous glands, and inflammatory cell infiltration at the wall and the periductal tissue [21]. We identified a similar phenomenon through cholangioscopy examination, where tissues surrounding the stricture were predominantly hyperplastic fibrous tissues with rare big neovascularization, especially in cases of membranous stricture. The use of CHFNKE with blend current in the incision or excision of a proliferative fibrous connective tissue layer rarely causes bleeding. However, there were 2 cases of intraoperative hemorrhage in the balloon group for the large tension mechanism, which were cured by choledochoscopic needle-knife electrocoagulation. Although the average operating time in mixing group was longer than electrotomy group or balloon group (*P* < 0.05), in order to get better efferect and induce the risk of bleeding, CHFNKE should be comblined with BD. At the same time of resolving of AS, the tissue damage caused by CHFNKE or BD can lead to the proliferation of scar tissue. So there should be long-term placement of internal/external biliary drainage to prevent anastomotic restricture.

In our study, among the three groups, the mean period of drainage catheter placement was 6.3 ± 0.7 months, 6.5 ± 0.6 months, and 6.1 ± 0.4 months and there was no obvious difference between each other. The four-year recurrence rate was 26.3 % (5/19), 28.5 % (2/7), and 16.7 % (2/12) in electrotomy group, balloon group and mixing group, respectively. The data revealed that there were no obvious differences between the CHFNKE and the balloon groups, however, when compared with the former two groups, the effect in the mixing group resulted in a significant improvement. This is because membranous stricture is easy to resect with CHFNKE, but balloon dilatation is applied to tubular stricture. Membranous stricture was defined as a layer of fibrous membrane attached to the bile duct wall and a narrow opening in the center of fibrous membrane. Tubular stricture was defined as a narrow lumen of bile duct more than 0.5 cm. Therefore, the combination of CHFNKE with balloon dilation can be used for the treatment of all types of membranous stricture.

This study investigated the feasibility of long-term internal/external drainage following CHFNKE, BD, or CHFNKE combined with BD, in terms of the technical complications and long-term outcomes of patients with anastomotic stricture after Roux-en-Y hepaticojejunostomy. Our results indicate that this complementary medical procedure has great potential as a new approach for the treatment of anastomotic stricture. However, caution must be exerted, as this exciting outcome needs further confirmation by following randomized-controlled trial. Meanwhile, the role of CHFNKE in treating anastomotic stricture and its mechanisms in the underlying tissue remodeling should be further investigated in animal models.

## Conclusions

In conclusion, this cross-sectional study shows choledochoscopic high-frequency needle-knife electrotomy is both feasible and safe in the treatment of anastomotic stricture after Roux-en-Y hepaticojejunostomy. In order to induce the recurrence, the combination of choledochoscopic electrotomy concurrent with balloon dilation should be recommended.

## Ethics approval and consent to participate

The study was approved by the Conduct of Human Ethics Committee of Affiliated Zhongshan Hospital of Dalian University. All patients have signed an informed consent form before inclusion.

## Consent for publication

Not applicable.

## Availability of data and materials

All the data supporting our findings is contained within the manuscript.

## Abbreviations

AS: anastomotic stricture
BD: balloon dilation
CHFNKE: choledochoscopic high-frequency needle-knife electrotomy
CT: computerized tomography
LFTs: liver function tests
MRCP: magnetic resonance cholangiopancreatography
PTBD: percutaneous transhepatic biliary drainage
PTC: percutaneous transhepatic cholangiography
